# Overcoming acquired chemo-resistance to gemcitabine: implications from the perspective of multi-modal therapy including surgery for pancreatic cancer

**DOI:** 10.20517/cdr.2021.75

**Published:** 2021-09-07

**Authors:** Fuyuhiko Motoi

**Affiliations:** Department of Surgery I, Yamagata University, Yamagata 990-9585, Japan.

**Keywords:** Gemcitabine, chemo-resistance, pancreatic cancer, multi-modal therapy, surgery

## Abstract

Gemcitabine has been used as a key drug for the treatment of pancreatic ductal adenocarcinoma. Although surgery remains the mainstay for cure of this lethal disease, the effect is quite limited, even for resectable disease, if there is no collaboration with chemotherapy. In the cases with unresectable disease, conversion surgery after a favorable response to chemotherapy might show encouraging results. Potentiation of chemotherapeutic agent is urgently needed in almost all stages of pancreatic cancer. Further efforts must be paid on overcoming chemo-resistance by understanding tumor diversity and developing biomarkers that follow recent success of modified conventional agents by drug delivery technology.

Saiki *et al.*^[1]^ successfully described a fine, exciting, and comprehensive review about gemcitabine metabolism from the perspective of overcoming acquired chemo-resistance in cancer cells, which was published in *Cancer Drug Resistance*. Gemcitabine has been widely used as a key drug for the treatment of pancreatic ductal adenocarcinoma (PDAC), which is globally one of the most lethal diseases with an extremely low survival rate, as they mentioned in their manuscript^[1]^. Although PDAC is known to be a chemo-resistant cancer with a low response rate to several conventional agents, recent advances in drug delivery technology have shown that improved delivery of conventional anti-cancer agents, such as paclitaxel (modified to Nab-Paclitaxel^[2]^) or irinotecan (modified to Nanoliposomal irinotecan^[3]^) significantly increased their clinical efficacy in cancer treatment^[2,3]^. Although gemcitabine is an effective agent, further improvement in its activity to obtain the similar success as with the above drugs, might be achieved, as the authors precisely described in their review^[1]^. This commentary is aimed to clarify the implication of overcoming chemo-resistance in PDAC treatment from the perspective of multi-modal therapy including surgery to improve the survival outcome of the lethal disease.

Although the mainstay of treatment that provides the only chance for cure of PDAC is curative surgery, the effect of surgery alone on survival is quite limited, as was unfortunately confirmed by the discouraging results of several randomized control trials evaluating the effect of extended resection^[4]^. The fact that most patients with PDAC after curative resection experience early distant relapse^[5]^ indicates that there might be occult micro-metastases outside the surgical field at the time of surgery. Even with curative surgery for resectable PDAC, the serum tumor marker carbohydrate antigen 19-9 (CA19-9), often shows sustained elevation after resection, followed by metastatic relapse in the liver (outside of the planned surgical field)^[6,7]^. To improve survival outcomes, surgical resection of the primary tumor load should be planned, taking appropriate measures to reduce micrometastases. Combining effective systemic chemotherapy and surgery, including the concepts of adjuvant and neoadjuvant therapy, has improved the survival outcomes of surgery for PDAC^[8]^. Since several solid studies support the significant survival benefit of adjuvant chemotherapy, adjuvant treatment has become the gold standard for patients with curatively resected PDAC^[9-12]^. Recently, the Prep-02/JSAP05 study^[13]^ revealed that neoadjuvant chemotherapy provided significant survival benefit and prolonged survival compared to upfront surgery, which has until recently been the gold standard approach for resectable PDAC^[14]^. Pooled analysis of randomized control trials also revealed the significantly longer disease-free survival and higher R0 resection rate obtained by neoadjuvant intervention^[15]^. Moreover, even in cases with unresectable PDAC at the initial diagnosis, conversion surgery after a favorable response to chemotherapy showed encouraging results^[16]^. Total neoadjuvant therapy, which consists of long-term multi-drug systemic chemotherapy and radiotherapy followed by surgery, also demonstrated impressive survival outcomes for locally advanced unresectable PDAC^[17]^. The progress of a multidisciplinary treatment strategy including surgery for PDAC relies on the recent advances in systemic chemotherapy for PDAC. Even in PDAC cases with a poor prognosis^[17,18]^, development of key new drugs, as suggested by Saiki *et al.*^[1]^, might overcome the dismal prognosis by improving both chemotherapy and multidisciplinary strategies including surgery. Further efforts to improve the efficacy of chemotherapeutic agents are essentially needed to confirm the progress of the strategy. Recent advances in the understanding of tumor heterogeneity by gene-expression profiling might uncover principles with clinical implications, finding a key to improve the outcome^[19]^. Establishment of potential biomarkers for drug activity is also important to benefit specific treatment especially in PDAC with gemcitabine^[20]^.

Overall, I greatly appreciate this work by Saiki *et al.*^[1] ^and agree that further studies are needed for gemcitabine-based treatment to be included in personalized medicine tailored for numerous molecular therapeutic targets in multiple pathogenic pathways.

## DECLARATIONS

### Copyright

© The Author(s) 2021.

## References

[1] Saiki Y, Hirota S, Horii A (2020). Attempts to remodel the pathways of gemcitabine metabolism: recent approaches to overcoming tumours with acquired chemoresistance. Cancer Drug Resist.

[2] Von Hoff DD, Ervin T, Arena FP (2013). Increased survival in pancreatic cancer with nab-paclitaxel plus gemcitabine. N Engl J Med.

[3] Wang-gillam A, Li C, Bodoky G (2016). Nanoliposomal irinotecan with fluorouracil and folinic acid in metastatic pancreatic cancer after previous gemcitabine-based therapy (NAPOLI-1): a global, randomised, open-label, phase 3 trial. Lancet.

[4] Jang JY, Kang JS, Han Y (2017). Long-term outcomes and recurrence patterns of standard versus extended pancreatectomy for pancreatic head cancer: a multicenter prospective randomized controlled study. J Hepatobiliary Pancreat Sci.

[5] Matsumoto I, Murakami Y, Shinzeki M (2015). Proposed preoperative risk factors for early recurrence in patients with resectable pancreatic ductal adenocarcinoma after surgical resection: a multi-center retrospective study. Pancreatology.

[6] Motoi F, Rikiyama T, Katayose Y, Egawa S, Unno M (2011). Retrospective evaluation of the influence of postoperative tumor marker status on survival and patterns of recurrence after surgery for pancreatic cancer based on RECIST guidelines. Ann Surg Oncol.

[7] Motoi F, Murakami Y, Okada KI, Multicenter Study Group of Pancreatobiliary Surgery (MSG-PBS) (2019). Sustained elevation of postoperative serum level of carbohydrate antigen 19-9 is high-risk stigmata for primary hepatic recurrence in patients with curatively resected pancreatic adenocarcinoma. World J Surg.

[8] Parmar A, Chaves-Porras J, Saluja R (2020). Adjuvant treatment for resected pancreatic adenocarcinoma: a systematic review and network meta-analysis. Crit Rev Oncol Hematol.

[9] Oettle H, Neuhaus P, Hochhaus A (2013). Adjuvant chemotherapy with gemcitabine and long-term outcomes among patients with resected pancreatic cancer: the CONKO-001 randomized trial. JAMA.

[10] Neoptolemos JP, Palmer DH, Ghaneh P (2017). Comparison of adjuvant gemcitabine and capecitabine with gemcitabine monotherapy in patients with resected pancreatic cancer (ESPAC-4): a multicentre, open-label, randomised, phase 3 trial. Lancet.

[11] Uesaka K, Boku N, Fukutomi A (2016). Adjuvant chemotherapy of S-1 versus gemcitabine for resected pancreatic cancer: a phase 3, open-label, randomised, non-inferiority trial (JASPAC 01). Lancet.

[12] Conroy T, Hammel P, Hebbar M, Canadian Cancer Trials Group and the Unicancer-GI-PRODIGE Group (2018). FOLFIRINOX or gemcitabine as adjuvant therapy for pancreatic cancer. N Engl J Med.

[13] Motoi F, Kosuge T, Ueno H, Study Group of Preoperative Therapy for Pancreatic Cancer (Prep) and Japanese Study Group of Adjuvant Therapy for Pancreatic cancer (JSAP) (2019). Randomized phase II/III trial of neoadjuvant chemotherapy with gemcitabine and S-1 versus upfront surgery for resectable pancreatic cancer (Prep-02/JSAP05). Jpn J Clin Oncol.

[14] Unno M, Motoi F, Matsuyama Y (2019). Randomized phase II/III trial of neoadjuvant chemotherapy with gemcitabine and S-1 versus upfront surgery for resectable pancreatic cancer (Prep-02/JSAP-05). J Clin Oncol.

[15] Birrer DL, Golcher H, Casadei R (2021). Neoadjuvant therapy for resectable pancreatic cancer: a new standard of care. Pooled data from three randomized controlled trials. Ann Surg.

[16] Satoi S, Yamaue H, Kato K (2013). Role of adjuvant surgery for patients with initially unresectable pancreatic cancer with a long-term favorable response to non-surgical anti-cancer treatments: results of a project study for pancreatic surgery by the Japanese Society of Hepato-Biliary-Pancreatic Surgery. J Hepatobiliary Pancreat Sci.

[17] Murphy JE, Wo JY, Ryan DP (2019). Total neoadjuvant therapy with FOLFIRINOX in combination with losartan followed by chemoradiotherapy for locally advanced pancreatic cancer: a phase 2 clinical trial. JAMA Oncol.

[18] Philip PA, Lacy J, Portales F (2020). Nab-paclitaxel plus gemcitabine in patients with locally advanced pancreatic cancer (LAPACT): a multicentre, open-label phase 2 study. Lancet Gastroenterol Hepatol.

[19] Adam RS, Blomberg I, Ten Hoorn S, Bijlsma MF, Vermeulen L (2021). The recurring features of molecular subtypes in distinct gastrointestinal malignancies-A systematic review. Crit Rev Oncol Hematol.

[20] Randazzo O, Papini F, Mantini G (2020). "Open Sesame?": biomarker status of the human equilibrative nucleoside transporter-1 and molecular mechanisms influencing its expression and activity in the uptake and cytotoxicity of gemcitabine in pancreatic cancer. Cancers (Basel).

